# Development, implementation and evaluation of Australia’s first national continuing medical education program for the timely diagnosis and management of dementia in general practice

**DOI:** 10.1186/s12909-018-1295-y

**Published:** 2018-08-10

**Authors:** Heike Schütze, Allan Shell, Henry Brodaty

**Affiliations:** 1 School of Health and Society, University of Wollongong, Wollongong, NSW 2522 Australia; 2 Dementia Centre for Research Collaboration, UNSW Australia, Level 3, AGSM Building, Sydney, 2052 Australia

**Keywords:** Dementia, Alzheimer’s disease, Continuing medical education, Primary care, General practice

## Abstract

**Background:**

Dementia is the second leading cause of death in Australia. Over half of patients with dementia are undiagnosed in primary care. This paper describes the development, implementation and initial evaluation of the first national continuing medical education program on the timely diagnosis and management of dementia in general practice in Australia.

**Methods:**

Continuing medical education workshops were developed and run in 16 urban and rural locations across Australia (12 were delivered as small group workshops, four as large groups), and via online modules. Two train-the-trainer workshops were held. The target audience was general practitioners, however, international medical graduates, GP registrars, other doctors, primary care nurses and other health professionals were also welcome. Self-complete questionnaires were used for the evaluation.

**Results:**

Of 1236 people (GPs, other doctors, nurses and other health professionals) who participated in the program, 609 completed the full program (small group workshops (282), large group workshops (75), online modules (252)); and 627 elected to undertake one or more individual submodules (large group workshops (444), online program (183)). Of those who completed the full program as a small group workshop, 14 undertook the additional Train-the-trainer program. 76% of participants felt that their learning needs were entirely met and 78% felt the program was entirely relevant to their practice.

**Conclusion:**

Continuing medical education programs are an effective method to deliver education to GPs. A combination of face-to-face and online delivery modes increases reach to primary care providers. Train-the-trainer sessions and online continuing medical education programs promote long-term delivery sustainability. Further research is required to determine the long-term knowledge translation effects of the program.

## Background

Dementia or Major Neurocognitive Disorder is a global public health priority [1] and is a leading cause of death in Australia [2]. The prevalence of dementia increases with age, doubling every 5 years between the ages of 60 and 85 years, and in 2017 over 413,100 Australians experienced some form of dementia; this is expected to rise to around 536,000 by 2025 and 1.1 million by 2056 [3].

The timely diagnosis of dementia forms the basis for effective management, offering consumers and their families the opportunity of treatment, support, and to plan for the future [4, 5], which can relieve the significant psychological distress that may be experienced by patients and their carers [6]. Over 85% of Australians visit their family physician or general practitioner (GP) each year [7]. As the usual first point of contact for patients, GPs have a key role in symptom recognition, assessment and referral, and are well placed to provide continuing care coordination and support. However, GPs do not readily diagnose dementia during routine practice visits, and there is evidence that dementia is under-diagnosed in primary care, with an estimated 60% of cases remaining undiagnosed [8–11]. This results in missed opportunities to treat symptoms, reduced planning time and reduced access to community resources and support [12, 13].

Although the need for GP education [14] and the need to improve the primary care response to dementia has been recognised for some time [12, 15–17], significant barriers to timely diagnosis persist including: a lack of GP confidence or training, limited time, a perceived lack of the need to determine a specific diagnosis, a lack of a recognised time-efficient screening tools, and difficulties in differentiating normal ageing from dementia [18–23]. Ensuring GPs are aware of early detection issues, understand basic screening techniques, and are aware of the treatments and community support options available for both dementia patients and their carers [24] may assist in addressing these issues.

Continuing medical education (CME) programs, are an effective means to deliver dementia educational [25] and in improving dementia detection rates [6, 26]. This paper describes the development, implementation and initial evaluation of the first Australian national CME program to assist general practitioners and primary care nurses to better understand, diagnose and manage dementia and/or mild to moderate cognitive impairment, in primary care.

## Methods

### Participants and setting

The target audience for the educational material was general practitioners, however, international medical graduates, GP registrars, other doctors, primary care nurses and other health professionals were also welcome.

### Development of the educational intervention

The CME training content was developed based on, *Dementia: 14 essentials of assessment and care planning* [4], and *Dementia: 14 essentials of management* [5]; previous materials developed for workshops (see acknowledgements); and consultation with an expert Steering Committee comprised of consumers, GPs, and representatives from the Dementia Centre for Research Collaboration, Dementia Training and Study Centre, Alzheimer’s Australia and GP Victoria, who provided critical review of the material once it was written.

The face-to-face program consisted of four 1.5 h sessions facilitated by a GP, assisted by specialist geriatricians and/or old age psychiatrists involved in the management of patients living with dementia. In addition, the government funded Dementia Behaviour Management Advisory Service, Alzheimer’s Australia and local geriatricians were invited as guest panel members and to provide information on available local services. Each session commenced with a 10 min video segment that followed the two-year progress of three people living with dementia. This was followed with didactic content around the specific topic focus of the session (see Table 1), and the remainder of the session was dedicated to case studies and discussions of participants’ case presentations, care gaps and the guidelines for managing cases. Topics covered included: understanding different types of dementia; screening and screening tools for dementia; barriers to diagnosis; use of pharmacological and non-pharmacological methods to manage behavioural and psychological symptoms of dementia; the impact of comorbidities on dementia; carer management education, support and referral; and legal issues with dementia. The materials were then adapted and packaged to suit the online modules and Train-the-Trainer sessions. A copy of the training materials are available [27].

**Table 1:** Breakdown of CME sessions (based on 1.5 h face-to-face session)

Step	Time	Content covered
1	10 min	Video introduction following the real-life progress over two years of three patients living with dementia and their families
2	30 min	Didactic presentation
		Session 1. Diagnosis, tests, scans and new biomarkers
		Session 2. Physical activity, positive lifestyle and risk factors
		Session 3. Complications and behavioural changes
		Session 4. The carer as the patient and legal issues
3	30 min	Participants review and discuss the case studies, their own case presentations, and discuss guidelines for management and care gaps
4	10–15 min	Questions and answers
5	5 min	Evaluation

*CME* Continuing medical education

The program had five broad learning objectives:

- To increase awareness of the current clinical guidelines for the diagnosis and management of dementia in general practice;
- To improve diagnostic acumen with respect to cognitive impairment;
- To identify factors in both the practitioner and the patient that may be potential barriers to the diagnosis of dementia;
- To understand the impact of a patient with dementia on family and carers and the community support and services that are available;
- Patient Safety - Implement a system in the practice using screening tools for all patients over 75 years in whom there is a concern about memory problems, for both cognitive impairment and depression.

### Implementation of the educational intervention

#### CME points

The face-to-face workshops and online modules were accredited as CME activities by the Royal Australian College of General Practitioners (RACGP). All RACGP-approved CME activities are eligible for CME points with the Australian College of Rural and Remote Medicine (ACRRM), therefore separate ACRRM accreditation was not applied for to reduce the administrative burden on the Timely Diagnosis of Dementia team. Table 2 shows the breakdown of the sessions offered and the corresponding CME points.

**Table 2:** Session duration and RACGP CME Point allocation for workshops and online modules

Delivery Method	Session Title	Time (hours)	CME Points
Large group workshop (GPCE)	14 Essentials(Brodaty et al., 2013a; b)	1.5	2
	S1: Diagnosis, Tests, Scans and New Biomarkers	1.5	2
	S2: Physical Activity, Positive Lifestyle and Risk Factors	1.5	2
	S3: Complications and Behavioural Changes	1.5	2
	S4: The Carer as the Patient and Legal Issues	1.5	2
	OR Timely Diagnosis of Dementia in General Practice (i.e. any 4 sessions)	6	40
Small group workshops	Timely Diagnosis of Dementia in General Practice (see S1-S4 above)	6	40
Online	Recognising Dementia	1	2
	Diagnosis of Dementia	1	2
	Screening and Case-finding	1	2
	Developing a Plan for Management	1	2
	Recognising and Managing BPSD and Physical Comorbidities	1	2
	Carer, Legal and End of Life Issues	1	2
	OR Timely Diagnosis of Dementia in General Practice (i.e. all six modules)	6	40
Train-the-trainer	Timely Diagnosis of Dementia in General Practice	6	40

*CME* Continuing medical education, *GPCE* General Practice Conference and Exhibition, *RACGP* Royal Australian College of General Practitioners

Participants wishing to apply for the maximum 40 CME points were required to undertake a predisposing activity prior to undertaking the program and a reflection activity after completion. Predisposing activities were designed to help participants think about the diagnosis and management of dementia in their practice and included pre-readings and writing a brief description of a case in their practice. Reinforcing activities required participants to address how they would apply their learnings to strengthen their practice. A copy of these are available [27].

#### CME activities

These were delivered as small group and large group face-to-face workshops and online modules as detailed below. Participants were not charged a fee for any of these activities.

##### Small group face-to-face workshops

These were held in urban and rural locations across Australia from November 2012 to November 2013. Medicare Locals in each state were contacted to gauge their level of interest in having a dementia CME program run in their area and to see if they were willing to send out a flyer advertising the availability of the workshop to all general practices in their catchment area. (Medicare Locals were 61 regional organisations across Australia which coordinated general practice and other health care services for a geographic area. They were organised into metro, regional and rural peer groups based on Socio-Economic Indexes for Areas and Remoteness Area categories [28]. Medicare Locals have since been replaced by Primary Health Networks). Once a Medicare Local was engaged, they were asked to provide suggestions for suitable local venues to run the CME workshop. Participants registered for workshops directly with the Timely Diagnosis and Management of Dementia project team. Medicare Locals were encouraged to engage further with the CME workshop, and some became more involved with promoting the workshop and attending on the day, whereas others ended their involvement with distributing the CME flyer to their general practices and suggesting local venues. The respective state and territory Alzheimer’s Australia organisations were contacted in order to coordinate a community-based awareness activity to occur in association with each workshop.

##### Large group face-to-face workshops

These were held at the General Practitioner Conference and Exhibition (GPCE) in the capital cities of Sydney, Melbourne, Perth and Brisbane. The GPCE meetings attract large numbers of GPs and therefore provided an opportunity to extend the reach of the workshops to more GPs. Participants registered directly with the conference provider and the conference provider charged a registration fee to attend the conference. There was no additional charge for participants to attend the workshops. The Timely Diagnosis and Management of Dementia project did not receive payment to deliver the workshops at the GPCE. The respective state and territory Alzheimer’s Australia organisations were contacted in order to coordinate a community-based awareness activity to occur in association with each GPCE.

##### Online modules

An external provider was contracted to help convert the material from the small group workshops to an online web-based version of the CME and to host the modules on their website. The modules went live in June 2013.

##### Train-the-trainer face-to-face workshops

The Timely Diagnosis of Dementia project team directly invited participants who had completed the 40 point CME face-to-face workshops in New South Wales or Victoria to participate in a Train-the-trainer Workshop, which qualified as an additional 40 CME point activity.

### Evaluation of the educational intervention

People who attended a face-to-face workshop or completed one or more online modules were invited to rate the degree to which their learning needs were met and the relevance of the program to their practice via an Evaluation Form that was included in the learning materials package. Forms could be returned when convenient. Participants were also provided a free text field and asked to suggest ways the activity could have been improved. The responses of the free text field were coded thematically.

## Results

Of the 1236 people who participated, 609 completed the full 40 point CME activity (small group workshops (282), large group workshops (75), online modules (252)), and 627 participants complete one or more 2 point CME activities (large workshops (444), online program (183)) (see Table 3). Fourteen people who completed the full 40 point CME program in the small group workshops, undertook the additional Train-the-trainer program, which qualified as an additional 40 CME point activity.

**Table 3:** Participants and evaluations by location, delivery method and number of CME points, Nov 2012 - Dec 2013

Workshop City (State)	Metro/Rural	40 Point CME		2 Point CME	
		Participants	Evaluations^a^	Participants	Evaluations^a^
Adelaide North (South Australia)	metro	23	20		
Ballina (New South Wales)	rural	25	8		
Bendigo (Victoria)	rural	13	1		
Darwin (Northern Territory)	metro	47	38		
Deakin (Australian Capital Territory)	rural	23	22		
Dubbo (New South Wales)	rural	39	7		
Geelong (Victoria)	rural	16	8		
Launceston (Tasmania)	rural	29	16		
Leederville (Western Australia)	rural	15	13		
Lithgow (New South Wales)	rural	13	3		
Preston (Victoria)	rural	15	15		
Traralgon (Victoria)	rural	24	15		
GPCE - Sydney (New South Wales)	metro	24	22	156	258^a^
GPCE - Perth (Western Australia)	metro	14	14	99	209^a^
GPCE - Brisbane (Queensland)	metro	15	15	76	144^a^
GPCE - Melbourne (Victoria)	metro	22	19	113	189^a^
Online	ND	252	252	183	183^a^
Total		609	488	627	983^a^
TTT Preston (Victoria)	rural	11	11		
TTT Sydney (New South Wales)	metro	3	0		
Total		14	11		

*CME* Continuing medical education, *TTT* Train-the trainer; \: Not applicable; ND: data not available ^a^participants could undertake more than one 2 point CME activity and could therefore provide more than one evaluation

A total of 1471 evaluations were received rating the degree to which learning needs were met and the relevance of the CME activity to practice (see Table 3). People who undertook the 2 CME point activities could undertake more than one activity and could therefore provide more than one evaluation. The activities were well regarded in that most participants (76%) felt that their learning needs were entirely met and 78% stated that the activity was entirely relevant to their practice (Table 4).

**Table 4:** Participant ratings that learning needs were met and relevance to practice, Nov 2012 - Dec 2013

Role	Degree to which learning needs were met					Degree of relevant to practice				
	Not met	Partially met	Entirely met	Not answered	Total	Not met	Partially met	Entirely met	Not answered	Total
GP	1	64	398	3	466	0	47	407	12	466
	0.1%	4.4%	27.1%	0.2%	31.7%	0.0%	3.2%	27.7%	0.8%	31.7%
IMG	0	1	38	0	39	0	0	39	0	39
	0.0%	0.1%	2.6%	0.0%	2.7%	0.0%	0.0%	2.7%	0.0%	2.7%
Doctor (other)	0	4	16	0	20	1	5	14	0	20
	0.0%	0.3%	1.1	0.0%	1.4%	0.1%	0.4%	1.0%	0.0%	9.5%
Nurse	6	44	83	0	133	7	37	89	0	133
	0.4%	3.1%	5.9%	0.0%	9.0%	0.5%	2.5%	6.1%	0.0%	9.0%
Other HP	0	7	35	0	42	0	8	34	0	42
	0.0%	0.5%	2.4%	0.0%	2.9%	0.0%	0.5%	2.3%	0.0%	2.9%
Role unknown	9	182	572	8	771	8	113	627	23	771
	0.6%	12.3%	38.9%	0.6%	52.4%	0.5%	7.7%	42.6%	1.6%	52.4%
Total	16	302	1142	11	1471	16	210	1210	35	1471
	1.0%	20.5%	77.6%	0.7%	100.0%	1.1%	14.3%	82.3%	2.4%	100.0%

*GP* General Practitioner, *HP* Health Professional, *IMG* International Medical Graduate

Of the answers received in the free-text comments on how the activity could be improved, the highest response (34%) across all delivery methods was that the activity did not need any changes:

*“Could not realistically improve this - a quality event.”* Rural GP

Although the activity was directed to GPs, non-GP participants also found the material useful:

“*As an OT [Occupational Therapist] some sections were too detailed for me (such as the medications) but still interesting - no comments to improve”* Online

*“Extremely well presented. Appropriate for my PN [Practice Nurse] /Community Care role”* Nurse

A variety of responses of how the activity could be improved were received (a summary of coded response categories is provided in Table 5). Some participants suggested that the workshops or online modules could be made more interactive and have more group work and discussion:

*“Less lecture style, more discussion and group work”* Rural GP

*“More small group discussion - might have learned more from colleagues - practical advice”* Urban GP

**Table 5:** Participant feedback on how the activity could be improved, Nov 2012 - Dec 2013

	GP	IMG	Doctor (other)	Nurse	Other HP	Role unknown	Total
Activity designed for GPs not nurses	0	0	0	7	0	0	7
Activity too long/too much information	14	2	0	0	2	15	33
Better use of practice nurses	1	0	0	0	0	0	1
Excellent / No improvements required	220	29	11	48	27	170	505
Have smaller workshops	4	0	0	0	0	0	4
Improve audio visual or workbook output quality	20	1	1	2	1	2	27
Include guest speaker from Alzheimer’s Australia	2	0	0	0	0	0	2
Less repetition	10	0	0	0	0	8	18
Presenter needs to stay on topic	0	0	0	0	0	9	9
Provide more case studies and/or examples	35	0	4	3	0	1	43
Provide more information on local services/referral services	15	1	0	2	0	2	20
Provide more information on medico-legal issues	3	0	0	0	0	0	3
Provide more information on pathology/diagnostic tools	6	0	0	1	0	1	8
Provide more information on patient care/patient management	5	1	0	0	1	1	8
Provide more information on pharmacology	7	0	0	0	1	1	9
Provide more multiple choice questions	11	0	1	0	0	0	12
Provide more time/Increase length of workshop	8	1	0	2	0	12	23
Provide more reference materials	6	1	0	1	0	1	9
Provide more videos / interaction, less lecture/reading	29	1	3	2	0	1	36
Provide practical experience	3	0	0	0	0	3	6
Provide regular updates	5	0	0	0	0	0	5
Specialised geriatrician speaker needed	1	0	0	0	0	0	1
Split activity for clinicians and non-clinicians	1	0	0	1	0	0	2
Other	17	1	0	2	0	6	26
No comment provided	43	1	0	62	10	538	654
Total	466	39	20	133	42	771	1471

*GP* General Practitioner, *HP* Health Professional, *IMG* International Medical Graduate

Others felt that providing more case studies, examples or time to practice with colleagues would have been beneficial:

*“Practice in administering the screening tools, perhaps on each other”* Rural GP

*“More case studies”* Online

Some participants felt there was too much information to be covered and that the volume of information needed to be decreased or alternatively the allocated time should be increased:

*“If the time period of this workshop had been increased OR if in future its time increase to two day[s], it would be more helpful”* Rural GP

*“This topic has many facets and the needs are great - actually need more time”* Nurse

Some suggested that more information on local referral services could have been supplied:

*“Examples of management of the patients in local community (in Perth WA [Western Australia] for example); what services and agencies are available and access to them”* Rural GP

*“More reference to/exploration of local services as they relate to the topic”* Rural GP

Having local information was also highlighted in terms of the legal aspects of dementia management:

*“Just in regards to the legal aspect of management, good to have guidelines from VIC [Victoria] state as well”* Online

A criticism of the online delivery method was providing suggested answers in made the activity a soft learning exercise:

*“Not including suggested answers on same page as the question...”* Online

## Discussion

This paper describes the development, implementation and initial evaluation of Australia’s first national, freely available and accredited, CME program on the Timely Diagnosis and Management of Dementia in General Practice. Dementia education training has been shown to have a positive effect on health professionals’ knowledge, attitudes and the provision of patient care [29] and can improve effective screening through the use of assessment tools and referral to community resources [30]. As such, continuing medical education programs can provide channels for dementia care knowledge translation.

Multiple factors influence choice of CME programs, including relevance to area of practice, wanting to remain up to date, the educational value of the program, convenience in terms of time and location, and delivery method [31]. Online CME programs are increasing in popularity as they provide learning options to those in rural areas [32], those who are time poor [33], and appeal to younger GPs [33, 34]. Although online CME activities address this gap, face-to-face CME programs where participants can engage with peers and experts remain the most popular educational formats for Australian GPs [31].

There is a need to increase opportunities to expand the reach of dementia education programs to reach larger primary care audiences [30]. This opportunity was created by delivering dementia CME programs that could be accessed free of charge in both face-to-face workshops and self-directed online e-learning formats. Within 13 months, 1236 people completed the CME program, and an additional 839 participants had begun the online activities (612 for the full 40 CME point program, 227 for one or more of the 2 CME point programs). This demonstrated not only the capacity of the program to reach large numbers, but importantly the interest of GPs and other health professionals for this targeted dementia education.

A key consideration in developing any program is long-term sustainability. Sustainability to deliver future workshops was achieved by upskilling a pool of interested participants as future facilitators through the Train-the-trainer program. Alzheimer’s Australia provided funding for a further series of workshops; however, whether facilitators will be interested in delivering workshops in the future without grant funding is unknown. Once developed, online CME programs offer a sustainable method for delivering CME education programs to large numbers at a comparatively small cost compared to face-to-face workshops, and their feasibility should be explored further.

Face-to-face workshops on dementia are opportune times to run Alzheimer’s Australia provider-focused community-based awareness programs and Primary Health Networks (formerly Medicare Locals) play an important role in advocating training opportunities to GPs in their area.

As such, negotiations for collaborative events between different organisations need to commence as early as possible to navigate competing demands and timetabling issues between organisations and to maximise involvement.

### Strengths and limitations

This study has several strengths. To ensure the integrity of the program, the materials were reviewed by an expert steering committee, including GPs and dementia consumers. The materials comprised a mix of lectures by expert speakers, videos, case scenarios, toolkits and guidelines. Multimodal dementia education programs are reported to be most effective when multiple sources of information are combined with active participation and/or reflection [35] as was provided in the full 40 point CME program.

Limitations include that participants self-elected to participate in the program and may represent a group of motivated and interested individuals. The program was run in the last year of a triennium for CME points which may have influenced participation rates as many GPs may already have accumulated sufficient points. Information would need to be gathered from those who participated to understand why they elected to take part in the program, and from those who did not to understand barriers to participation.

The current evaluation is descriptive, describing participants’ ratings of whether the CME program addressed their learning needs and was relevant to their practice and well as suggestions for improvements. It is currently unknown if meeting the perceived educational needs of health care professionals translated to improved practice. The CME program will be further evaluated to determine if long-term sustainable knowledge translation with regards to diagnosing and managing patients with dementia has occurred as a result of the educational program.

## Conclusions

This was the first nationwide CME program in Australia on the timely diagnosis and management of dementia in primary care. CME programs are an effective method to deliver education to GPs and can provide channels for dementia care knowledge translation.

A combination of face-to-face and online delivery modes increases reach to primary care providers. Train-the-trainer sessions and development of online CME programs promote long-term sustainability. Further research is required to determine the long-term knowledge translation effects of CME programs.

## Abbreviations

ACRRM: Australian College of Rural and Remote Medicine
CME: Continuing medical education
GP: General practitioner
GPCE: General Practitioner Conference and Exhibition
HP: Health professional
IMG: International medical graduate
RACGP: Royal Australian College of General Practitioners
TTT: Train-the-trainer
